# The Edinburgh Consensus: preparing for the advent of disease-modifying therapies for Alzheimer’s disease

**DOI:** 10.1186/s13195-017-0312-4

**Published:** 2017-10-26

**Authors:** Craig W. Ritchie, Tom C. Russ, Sube Banerjee, Bob Barber, Andrew Boaden, Nick C. Fox, Clive Holmes, Jeremy D. Isaacs, Ira Leroi, Simon Lovestone, Matt Norton, John O’Brien, Jim Pearson, Richard Perry, James Pickett, Adam D. Waldman, Wai Lup Wong, Martin N. Rossor, Alistair Burns

**Affiliations:** 1 Centre for Dementia Prevention, University of Edinburgh, 9a Edinburgh BioQuarter, 9 Little France Road, Edinburgh, EH16 4UX UK; 2 Division of Psychiatry, Centre for Clinical Brain Sciences, University of Edinburgh, Edinburgh, UK; 3 Alzheimer Scotland Dementia Research Centre, University of Edinburgh, Edinburgh, UK; 4 Centre for Cognitive Ageing & Cognitive Epidemiology, University of Edinburgh, Edinburgh, UK; 5 Centre for Dementia Studies, Brighton and Sussex Medical School, Brighton, UK; 6 Old Age Faculty, Royal College of Psychiatrists, London, UK; 7 Alzheimer’s Society, London, UK; 8 Dementia Research Centre, Department of Neurodegenerative Disease, UCL, London, UK; 9 Faculty of Medicine, University of Southampton, Southampton, UK; 10 St George’s University Hospitals NHS Foundation Trust, London, UK; 11 Division of Neuroscience & Experimental Psychology, University of Manchester, Manchester, UK; 12 Department of Psychiatry, University of Oxford, Oxford, UK; 13 Alzheimer’s Research UK, Cambridge, UK; 14 Department of Psychiatry, University of Cambridge, Cambridge, UK; 15 Alzheimer Scotland, Edinburgh, UK; 16 Imperial College Healthcare NHS Trust, London, UK; 17 East and North Hertfordshire NHS Trust, Stevenage, UK; 18 Centre for Clinical Brain Sciences, University of Edinburgh, Edinburgh, UK

**Keywords:** Dementia, Alzheimer’s disease, Therapeutics, Disease-modification, Service redesign, Clinical trials

## Abstract

**Context:**

This commentary discusses the implications of disease-modifying treatments for Alzheimer’s disease which seem likely to appear in the next few years and results from a meeting of British experts in neurodegenerative diseases in Edinburgh. The availability of such treatments would help change public and professional attitudes and accelerate engagement with the prodromal and preclinical populations who might benefit from them. However, this would require an updated understanding of Alzheimer’s disease, namely the important distinction between Alzheimer’s disease and Alzheimer’s dementia.

**Consensus:**

Since treatments are likely to be most effective in the early stages, identification of clinically relevant brain changes (for example, amyloid burden using imaging or cerebrospinal fluid biomarkers) will be crucial. While current biomarkers could be useful in identifying eligibility for new therapies, trial data are not available to aid decisions about stopping or continuing treatment in clinical practice. Therefore, effective monitoring of safety and effectiveness when these treatments are introduced into clinical practice will be necessary to inform wide-scale use. Equity of access is key but there is a tension between universal access for everyone with a diagnosis of Alzheimer’s disease and specifying an eligible population most likely to respond. We propose the resources necessary for an optimal care pathway as well as the necessary education and training for primary and secondary care.

**Conclusion:**

The majority of current services in the UK and elsewhere would not be able to accommodate the specialist investigations required to select patients and prescribe these therapies. Therefore, a stepped approach would be necessary: from innovating sentinel clinical-academic centres that already have capacity to deliver the necessary phase IV trials, through early adoption in a hub and spoke model, to nationwide adoption for true equity of access. The optimism generated by recent and anticipated developments in the understanding and treatment of Alzheimer’s disease presents a great opportunity to innovate and adapt our services to incorporate the next exciting development in the field of dementia.

Driven by population ageing, dementia is now recognised as one of the greatest global public health challenges [1, 2]. The commonest aetiology of the dementia syndrome is Alzheimer’s disease. Currently, only symptomatic treatments for Alzheimer’s dementia are available and, as yet, the findings from therapeutic trials for drugs and biological agents to modify the course of Alzheimer’s disease before dementia develops are universally negative [3]. However, almost 100 treatments are currently being investigated, often targeting individuals earlier in the disease process [4], and very promising phase II work has been published [5]. Given ongoing research efforts, it seems likely that interventions—be they pharmacological or non-pharmacological multi-modal interventions—will be available in the near future for people diagnosed with prodromal dementia. This would fundamentally transform how the condition is perceived, diagnosed, and managed.

Even before the full dementia syndrome is present, Alzheimer’s disease may manifest as changes in biomarkers (‘preclinical’ disease) or minor cognitive symptoms (‘prodromal’ disease or ‘mild cognitive impairment’) [6, 7]. These pre-dementia stages of illness rely heavily on the understanding of an individual’s burden of disease measured using biomarkers found in cerebrospinal fluid (CSF) and through metabolic brain imaging. Consequently, any future selection of patients for treatment will require the ability to demonstrate such biomarker ‘abnormalities’ in contrast to the relatively simple clinical assessments needed to initiate cholinesterase inhibitors. This would require access to PET imaging or CSF sampling for patient selection for treatment rather than the relatively simple clinical assessments needed to initiate the current symptomatic treatments for Alzheimer’s disease. This is an exciting prospect but how such a transformation could be implemented in the UK National Health Service (NHS) is unclear.

## Consensus meeting

To address this lack of clarity, a group of academic clinicians from neurology, psychiatry, and neuroradiology disciplines and charity representatives met in Edinburgh to discuss how the NHS and other health care providers could respond to the future availability of a disease-modifying treatment for Alzheimer’s disease and what the role of biomarkers would be in the diagnostic process. The group discussed the implications for existing clinical services and the best way to adapt services to deliver the most appropriate and equitable access to such therapeutic advances. The overall purpose of the meeting and this report was to inform the predictable evolution in services, attitudes, and understanding by clarifying the changes to services, resource allocation, training, and public awareness that will be necessary. If implemented now, our message (Box 1) would also bring tangible benefits to people currently being referred to dementia services.

The group met once and was led through a pre-specified agenda by the three co-chairs MNR, AB, and CWR. The agenda covered the current use of and the science behind imaging and other biomarkers, the current pipeline of disease-modifying therapies for Alzheimer’s disease, and a review of current care pathways in the UK. The meeting was minuted by TCR and, before the conclusion of the meeting, the statements for this consensus statement were discussed and unanimously agreed by all present. Each co-author has contributed to the final drafting of this manuscript, thereby endorsing the views and proposals herein.

## Biomarkers for assessment and diagnosis

### Treatments are likely to be most effective in the early stages of Alzheimer’s disease

Alzheimer’s *disease* begins as early as mid-life and only manifests as Alzheimer’s *dementia* once the disease is at an advanced stage [8, 9]. Therefore, Alzheimer’s disease is generally far advanced by the time Alzheimer’s dementia is diagnosed, even at an “early” stage of dementia. There is an emerging consensus that optimal disease modification would be best achieved at earlier stages of the disease before dementia develops. Multiple failed phase III trials (most recently solanezumab and verubecestat [10]) seem to support this. Although stage of disease may have mediated trial failure, other possibilities include insensitive outcome measures, poor target engagement, heterogeneity of sample across multiple sites and languages, insufficient phase II data informing phase III confirmation, and the possibility that the specific targeting of the amyloid pathway is inadequate to generate clinical benefit.

**Fig. 1:**
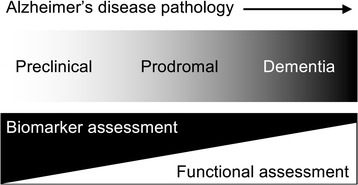
The continuum of Alzheimer’s disease pathology from the preclinical and prodromal stages to overt clinical dementia plus the relative importance of biomarker assessment and functional assessment at the different stages

Thus, future treatments will be initially offered to people with mid-stage Alzheimer’s disease but without symptoms sufficiently severe to merit a diagnosis of dementia, i.e. prodromal Alzheimer’s disease (Fig. 1). Eventually, treatments might be offered to people at an even earlier stage: people with biomarker evidence of Alzheimer’s disease but minimal or no symptoms, i.e. preclinical Alzheimer’s disease. There are several preclinical trials ongoing and this is the primary focus of the European Prevention of Alzheimer’s Dementia (EPAD) Consortium (http://ep-ad.org/) [11].

### Markers of cortical amyloidosis are fundamental

Treatment of Alzheimer’s dementia is currently ‘one size fits all’ with anyone with the clinical syndrome being considered for a cholinesterase inhibitor or memantine [12]. However, since early disease-modifying treatments for Alzheimer’s disease are likely to target amyloid aggregation—although tau-focused therapies are also in development—markers of cortical pathology such as amyloidosis will become central in assessing eligibility for new treatments. This drive towards molecular-based therapeutics will inevitably lead to molecular-based diagnostics. This would move the field towards precision medicine, allowing us to offer targeted treatments to individuals with cortical amyloidosis with the prospect of even greater precision in the future.

Therefore, markers of cortical pathology such as amyloidosis will become central in diagnosing Alzheimer’s disease—particularly at a preclinical stage—and in assessing eligibility for the new treatments. All current trials enrich the clinical sample recruited for the presence of cortical amyloidosis and it is highly likely that these selection criteria will be used to access the treatment in clinical practice.

Cerebral amyloidosis can be measured directly using amyloid PET imaging or indirectly in CSF. There is high concordance between these diagnostic approaches [13], so local availability, cultural factors, and staff experience will determine the choice between the two.

### The use of biomarkers for personalising intervention and for gauging response

In a research setting, PET-Amyloid Imaging, CSF Aβ_42_, tau, and p-tau are useful biomarkers to differentiate people with Alzheimer’s dementia from controls [14]. However, the current utility of these biomarkers in a clinical context, given the low specificity of these tests (e.g. CSF Aβ) for Alzheimer’s dementia in the elderly and the inconsistent quality of the evidence base is less clear [15, 16]. Eligibility criteria for new disease-modifying treatments would initially need to echo the entry criteria for the phase III trials for the treatment, all of which now include a marker of amyloid burden. Further enrichment of the clinical population using *APOE* status should only be recommended in clinical practice where there was a demonstrable pharmacogenetic effect noted in the treatment’s licensing trial (either for efficacy or safety) unless new evidence emerges for its utility in risk prediction models.

**Fig. 2:**
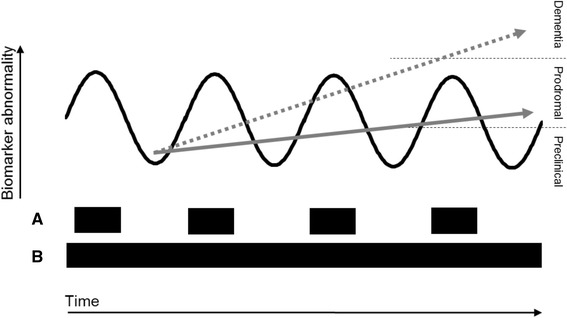
Hypothetical model of intervention with a disease-modifying treatment for Alzheimer’s disease. The curved line depicts a biomarker reflecting a specific drug target which responds to treatment. **a** depicts the courses of mechanism-specific drug(s). **b** depicts the accompanying tailored risk factor intervention and advice. The *dotted grey line* indicates the disease course without treatment—progressive functional deterioration. The *solid grey line* indicates the disease course with treatment—slower deterioration and better functional outcome, i.e. the delay of onset of clinical dementia. Adapted from Hampel et al. [12]

Early disease-modifying treatments will probably slow, rather than reverse, neurodegeneration and so on-going treatment may be required to maintain this effect. Treatment might be lifelong and last several decades. However, there are currently no reliable and validated patient-related outcome instruments for the early stages of the disease. To ensure ongoing clinical and cost-effectiveness, explicit and early stopping criteria would be important, which will require a surrogate biomarker response (Fig. 2). However, no biomarker currently studied is known to be suitable for this purpose and current trials (except DIAN-TU [17]) use intermediary biomarker phenotypes such as PET-Amyloid to determine if a treatment is futile. Furthermore, since clinical trials assess this at a group level and not for individuals, one would only be able to derive a probability of a lack of long-term success for an individual in clinical practice. Therefore, there is currently no reliable way to decide whether an individual should stop or continue other than on the grounds of safety. Embedding this outcome into phase II and III trials would facilitate greater appropriate access to therapies by minimizing inappropriate, ineffective use, but the limited duration of these trials (up to two years) would be a challenge.

Accessing the vast amount of observational data being gathered through programmes like the European Medical Information Framework (EMIF) and Dementias Platform UK (DPUK) will give an indication of the expected rate of changes in sub-populations—though direct comparison of these data with randomised, controlled trial data would be problematic from a statistical perspective. Phase IV testing—as advocated below—would address some of these issues as would the use of readiness cohorts (e.g. EPAD) that can use run-in data from the same population as the trial to help guide expected placebo declines in the trial population.

### The importance of phase IV testing

Substantial post-marketing research must be carried out before disease-modifying therapies are widely used and this should be co-produced by the public sector, charities, and industry alongside agreements on pricing and reimbursement that reflect optimum value for the NHS and patients. This would encompass accumulation of safety data as well as real world experience on mid- to long-term patient outcomes (including patient-related outcome measures), compliance, service impact, health economics, and longitudinal biomarker analysis to assist the establishment of a response biomarker. Generic phase IV protocols must be developed before these drugs are marketed so they can be safely and effectively introduced as widely as possible. This will involve close co-operation with bodies such as the National Institute for Health and Care Excellence (NICE), which approves the use of new treatments in the English and Welsh National Health Service and has major influence elsewhere. The establishment of better systems for gathering information from clinical practice would ease this phase IV testing if routinely collected electronic data contained phenotypic, clinical, and resource utilisation variables relevant to brain health in order to undertake large scale, standing phase IV work.

## Policy and professional development

### Equity of access is key

New treatments will initially be provided to patients in whom their use will be clinically and cost-effective and it is vital that when a new treatment becomes available it can be offered to everyone who is eligible. However, there is an inherent tension between specifying the eligible population through biomarkers (not to mention the uneven spread of tertiary-level expertise in dementia diagnosis and treatment) and equity of access—for example, for people of all ages, from all ethnic groups and from all localities. Patient and public engagement will be important to ensure that treatment provision is not perceived as inequitable.

In the first instance, prescription of disease-modifying treatments may only be possible in centres with capacity to undertake the partner diagnostic work as well as monitoring for safety issues and undertaking the phase IV work described above. Nevertheless, within these centres (which should act as regional hubs for prescribing) equity of access should be mandated and monitored, and access extended as quickly as possible. Close regional collaboration across NHS trust boundaries will be essential to this.

### Professionals will need specific training

There will need to be substantial education and training provided for primary and secondary care professionals about new disease-modifying treatment for Alzheimer’s disease. In primary care this would need to focus on early symptoms and risk factors to ensure timely referral whereas in secondary care it would cover the safe and effective use of biomarkers. Indeed, surveying current provision of dementia services and knowledge and attitudes should be done immediately to assess current service capacity and identify training needs. This could build on the existing Memory Services National Accreditation Programme (MSNAP) in England and Wales.

The UK has an established pool of clinical academics who already use these interventions in clinical trials as well as PET imaging and CSF biomarkers, either routinely or on selected patients in their clinical practice or research. However, further planning will be important and necessary to ensure this knowledge and expertise can be shared with all relevant staff groups across the NHS who will be operating from a diversity of clinical settings and backgrounds.

### This is an opportunity to reconfigure our services

Even without formal assessment of national and regional capacity, it is clear that increased access to neuroimaging and CSF analysis would be needed to support the additional diagnostic processes required for the appropriate targeting of new treatments. An agreed optimal care pathway would minimize idiosyncratic and inefficient practice. Initially rolled out in centres with relevant resources and expertise (forming a Brain Health Clinic Network), the resulting real world experience could be used to develop pathways for broader adoption throughout the NHS.

Dementia services are currently run under a predominantly psychiatric model of care with a symptomatic and palliative focus. However, a reconfigured service would require seamless collaboration between disciplines, patient groups, and specialties in order to expand the dementia-focussed clinical services to include an Alzheimer’s disease and pre-dementia service for cognitively healthier, younger patients. Indeed, engagement with other specialties who may be in contact with such patients because of other health conditions will be an important necessary development. The focus would be on maintaining brain health using risk reduction, resilience building, molecular-based diagnostics, and delivering complex pharmaceutical interventions.

## The optimal care pathway

### Who should be referred?

Many people—including those already diagnosed and not yet diagnosed with Alzheimer’s disease—would probably present to their GP when the marketing of a disease-modifying treatment was covered in the media. This has substantial resource implications for an already busy workforce. The number of specialists with relevant experience and appropriate facilities for diagnosis and treatment will represent important limitations.

Memory services currently see patients with later prodromal disease and early dementia but, over time, the profile of patients would also include preclinical and early prodromal populations. The future role of the GP as gatekeeper in general is uncertain [18] but direct access to a diagnostic and therapeutic service for Alzheimer’s disease may not be feasible or desirable. With treatment likely to involve complex eligibility assessment, including biomarkers, one could advocate GPs referring everyone with suspected Alzheimer’s disease to a specialist service. However, only a relatively small proportion of these people referred would meet the eligibility criteria for a new treatment. On the other hand, while many people currently present with moderate or severe dementia, in the future, hopefully the majority of people will be diagnosed much earlier, even in the prodromal/preclinical stages. Better cognitive tests for use in primary care with improved specificity and sensitivity for Alzheimer’s disease to help with referral decisions are currently in development. This would support primary care triage of patients into secondary care. However, until such tests exist, selection for treatment with disease-modifying therapies could only take place in specialist centres.

### What would an optimal specialist service require?

Disease-modifying therapies will be specific to Alzheimer’s disease and so there will be an even greater need to sub-type neurodegenerative diseases accurately. For this diagnostic process, access to a variety of imaging modalities to differentiate Alzheimer’s disease from other neurodegenerative conditions would be necessary—structural imaging (MRI, CT), FDG-PET, DAT, as well as amyloid and possibly tau PET (plus access to radiolabelled tracers)—plus neuroradiological and nuclear medicine expertise for the interpretation of these imaging modalities and their contextualisation alongside other assessments. Facilities for lumbar puncture would also be needed to ascertain cortical amyloidosis if PET imaging is not possible or acceptable to the patient.

The multidisciplinary team should include a specialist doctor; the skills of neurology, psychiatry, and geriatric medicine will all be relevant and should be represented. Furthermore, a new specialist nurse role could emerge, analogous to Macmillan nurses but with distinct skills from those already in existence. If pharmacogenetic testing (e.g. *APOE* ε4 status) is required for dose or subject selection, then embedding a genetic counselling service will also be required. Obviously, an appropriate setting to impart any diagnosis in a sensitive manner and to provide ongoing post-diagnostic information and support is vital. This service should be able to follow up people who have been commenced on a disease-modifying agent and should be able to escalate care in the case of adverse reactions during treatment administration. This might mean location in or near a general hospital.

A distinct approach for the preclinical, prodromal, and dementia stages of Alzheimer’s disease would be necessary. A comprehensive service could provide all these elements, but different expertise, skills, and care pathways are needed at different stages of disease. However, whether a broad, “one stop shop” is feasible or whether a tiered approach with primary, secondary (diagnostic), and tertiary (therapeutic) levels would be necessary is uncertain. In any case, transition through the service must be seamless. Furthermore, people with dementia often report that they wish services to be close to home.

That any new clinical service will have to keep pace with updates in diagnostic criteria for Alzheimer’s disease and other neurodegenerative conditions, it is noted that new criteria being proposed increasingly rely on biomarker evaluation which re-emphasises the need for clinical services to have access to and experience with biomarker testing as part of their clinical work-up.

### How many people might be eligible for a new treatment?

Based on current service capacity, gradual implementation, and variable take up of the new service, the likely number of eligible patients will initially be hundreds rising to tens of thousands of people within a few years as services adapt and increased public awareness helps case ascertainment in primary care. However, data to back up this estimate and further detailed modelling are needed to inform planning and resource allocation.

## Communication

### Disease-modifying treatments would change the way we all think about neurodegenerative diseases and dementia

There is much confusion in many people’s minds (including the public, clinicians, and policy makers) about dementia—for example, whether it is the same as Alzheimer’s disease. Irrespective of this, most people currently think of dementia as an untreatable, progressive condition affecting older people. The advent of a disease-modifying treatment would change this perception. The view that Alzheimer’s disease can only be diagnosed in the dementia stage of the illness must be challenged as the prognostic accuracy of pre-dementia disease models improves. This is familiar territory in other branches of medicine where treatments are commonly started before there are clinical signs or symptoms. A major societal benefit of the advent of disease-modifying drugs would be the end of the perception of Alzheimer’s disease as an incurable, inevitably fatal disease of older people and its replacement with a more optimistic position with a high likelihood of modification of the disease course.

The Edinburgh Consensus group recognised the need to advocate for the changes outlined above as well as continually reviewing progress in the UK and elsewhere as science advances, care models adapt, and therapies become available. These are truly innovative and optimistic times in the understanding and treatment of earlier Alzheimer’s disease. Modifying the disease course with multimodal interventions and personalised treatment plans—including risk reduction—will soon be possible. Working closely with European and global colleagues in the academic, clinical, charitable, and commercial sectors and with patient groups will help the largest number of people access the right therapies to prevent or significantly delay the onset of Alzheimer’s dementia.

## Box 1 Consensus message

- The advent of a disease-modifying treatment would represent a major positive advancement in the management of Alzheimer’s disease.
- This would change the perception of the illness to one that is treatable, which would mark a substantial change in long-held attitudes by the public and healthcare staff.
- Depending on the nature of the patient population for any new medication, redesign of services for Alzheimer’s disease is highly likely to be necessary.
- Healthcare systems will need to identify and engage with prodromal and preclinical populations who might benefit from such interventions. These people may not be in contact with health services or, if they are, this will not be because of Alzheimer’s disease.
- The implications of a disease-modifying treatment would amount to a paradigm shift in clinical approaches to Alzheimer’s disease, but one for which it is possible to prepare.
- Diagnosis, eligibility, and perhaps monitoring of treatment efficacy will require diagnostics to demonstrate evidence of cerebral amyloidosis as an example of precision medicine.
- Realistic planning is needed now to direct the evolution of services to optimise appropriate patient access and prepare protocols for phase IV testing of these treatments to inform real world practice and commissioning decisions.

## Box 1 Consensus message

- The advent of a disease-modifying treatment would represent a major positive advancement in the management of Alzheimer’s disease.
- This would change the perception of the illness to one that is treatable, which would mark a substantial change in long-held attitudes by the public and healthcare staff.
- Depending on the nature of the patient population for any new medication, redesign of services for Alzheimer’s disease is highly likely to be necessary.
- Healthcare systems will need to identify and engage with prodromal and preclinical populations who might benefit from such interventions. These people may not be in contact with health services or, if they are, this will not be because of Alzheimer’s disease.
- The implications of a disease-modifying treatment would amount to a paradigm shift in clinical approaches to Alzheimer’s disease, but one for which it is possible to prepare.
- Diagnosis, eligibility, and perhaps monitoring of treatment efficacy will require diagnostics to demonstrate evidence of cerebral amyloidosis as an example of precision medicine.
- Realistic planning is needed now to direct the evolution of services to optimise appropriate patient access and prepare protocols for phase IV testing of these treatments to inform real world practice and commissioning decisions.
