# Correction to: The Edinburgh Consensus: preparing for the advent of disease-modifying therapies for Alzheimer’s disease

**DOI:** 10.1186/s13195-018-0372-0

**Published:** 2018-07-30

**Authors:** Craig W. Ritchie, Tom C. Russ, Sube Banerjee, Bob Barber, Andrew Boaden, Nick C. Fox, Clive Holmes, Jeremy D. Isaacs, Ira Leroi, Simon Lovestone, Matt Norton, John O’Brien, Jim Pearson, Richard Perry, James Pickett, Adam D. Waldman, Wai Lup Wong, Martin N. Rossor, Alistair Burns

**Affiliations:** 1 Centre for Dementia Prevention, University of Edinburgh, 9a Edinburgh Bio Quarter, 9 Little France Road, Edinburgh, EH16 4UX UK; 2 Division of Psychiatry, Centre for Clinical Brain Sciences, University of Edinburgh, Edinburgh, UK; 3 Alzheimer Scotland Dementia Research Centre, University of Edinburgh, Edinburgh, UK; 4 Centre for Cognitive Ageing and Cognitive Epidemiology, University of Edinburgh, Edinburgh, UK; 5 Centre for Dementia Studies, Brighton and Sussex Medical School, Brighton, UK; 6 Old Age Faculty, Royal College of Psychiatrists, London, UK; 7 Alzheimer’s Society, London, UK; 8 Dementia Research Centre, Department of Neurodegenerative Disease, UCL, London, UK; 9 Faculty of Medicine, University of Southampton, Southampton, UK; 10 St George’s University Hospitals NHS Foundation Trust, London, UK; 11 Division of Neuroscience & Experimental Psychology, University of Manchester, Manchester, UK; 12 Department of Psychiatry, University of Oxford, Oxford, UK; 13 Alzheimer’s Research UK, Cambridge, UK; 14 Department of Psychiatry, University of Cambridge, Cambridge, UK; 15 Alzheimer Scotland, Edinburgh, UK; 16 Imperial College Healthcare NHS Trust, London, UK; 17 East and North Hertfordshire NHS Trust, Stevenage, UK; 18 Centre for Clinical Brain Sciences, University of Edinburgh, Edinburgh, UK

## Correction

Since the publication of our article [1], it has come to our attention that we failed to include information for two of the authors in the competing interests section. Craig Richie has declared potential competing interests with the following companies; Janssen, Eisai, Pfizer, Eli Lilly, Roche Diagnostics, Boeringher Ingleheim, Novartis, AC Immune, Ixico, Aridhia, Amgen, Berry Consultants, Lundbeck, Sanofi, Quintiles (IQVIA) and Takeda. Jeremy Issacs has declared potential competing interests with the following companies; Roche and Merck & Co. The full competing interests section for this article can be found below.

**Declarations**

**Competing interests**

Bob Barber has participated in an advisory panel for Novartis on one occasion and conducted a number of pharmaceutical sponsored commercial clinical trials in Alzheimer’s disease (including Lilly, Roche and Janssen). He has a national role for NIHR NHS relating to commercial studies in dementia. Nick Fox has received research support from AVID/Lilly and has advised GSK, Lilly, Novartis and Roche; he serves on a data monitoring committee for Biogen—all payments for these activities are to UCL. Clive Holmes has attended advisory boards for Lilly pharmaceuticals. Martin Rossor serves on a safety monitoring committee for Servier and has advised Merck on patient registries. Tom Russ works within NHS Scotland with the Scottish Neuroprogressive and Dementia Network on commercial studies in dementia. Craig Richie is the co-coordinator and academic lead for the EPAD (European Prevention of Alzheimer's Dementia) Project which has numerous commercial partners in keeping with the mechanisms of the European Union's Innovative Medicine's Initiative. These companies are: Janssen, Eisai, Pfizer, Eli Lilly, Roche Diagnostics, Boeringher Ingleheim, Novartis, AC Immune, Ixico, Aridhia, Amgen, Berry Consultants, Lundbeck, Sanofi, Quintiles (IQVIA) and Takeda. Jeremy Issacs is a Principal Investigator on clinical trials, outside of the submitted work, sponsored by both Roche and Merck & Co, who supply funding to his employing organisation to conduct the study. The remaining co-authors declare that they have no competing interests. All authors have completed the ICJME declaration of conflicts of interest.
